# Effective Adolescent Hand CRPS Type 1 Treatment Using Ketamine, Gabapentin, and Supraclavicular Nerve Block Catheter—A Case Report

**DOI:** 10.3390/children12121659

**Published:** 2025-12-07

**Authors:** Harshini Medikondu, Alexander Davit, Mihaela Visoiu

**Affiliations:** Department of Anesthesiology and Perioperative Medicine, University of Pittsburgh School of Medicine, UPMC Children’s Hospital of Pittsburgh, Pittsburgh, PA 15224, USA; medikonduh@upmc.edu (H.M.); alexander.davit@chp.edu (A.D.)

**Keywords:** allodynia, hyperalgesia, nerve block, regional anesthesia

## Abstract

A 15-year-old female developed refractory Complex Regional Pain Syndrome (CRPS) Type I of the left hand following metacarpal fixation. Conservative therapy and hand rehabilitation failed, resulting in persistent allodynia and functional loss. She was admitted for multimodal analgesia combining subanesthetic ketamine infusion, gabapentin, and a tunneled supraclavicular continuous nerve catheter delivering ropivacaine. Pain decreased from 7/10 at rest to 0/10 within 48 h. Allodynia has resolved, and motor function has fully recovered. The catheter was removed nine days later without complication, and pain remission persisted. This case demonstrates a safe and effective multimodal strategy for adolescent CRPS integrating central and peripheral desensitization mechanisms.

## 1. Introduction

Complex Regional Pain Syndrome (CRPS) is a chronic neuropathic pain disorder characterized by disproportionate pain, sensory disturbances, vasomotor changes, and functional impairment following tissue injury or surgery [1]. The underlying mechanisms involve peripheral inflammation, sympathetic dysregulation, and maladaptive neuroplasticity, leading to persistent pain and functional impairment.

Pediatric and adolescent CRPS, though less common than adult presentations, can be particularly disabling due to central sensitization and psychosocial vulnerability [2]. The condition may significantly impact physical function, academic participation, and emotional well-being. Early diagnosis and a multidisciplinary approach are critical for successful recovery.

Traditional management emphasizes exercise therapy, desensitization, and behavioral interventions. Exercise therapy and functional rehabilitation remain the cornerstone of treatment, as early mobilization can reverse sympathetic and cortical changes that perpetuate pain [3]. However, there is a subset of patients who present with severe symptomatology or fail to respond to initial conservative measures and require pharmacologic or interventional therapy to achieve desensitization and functional restoration.

Diagnostic clarity has improved through the use of the Budapest criteria, which provide a standardized clinical framework for identifying CRPS [4]. Pharmacologic options such as gabapentinoids, antidepressants, and NMDA receptor antagonists are often used to modulate neuropathic pain pathways. Ketamine has gained increasing use as an NMDA receptor antagonist that interrupts central sensitization and “wind-up” mechanisms [5].

Regional techniques provide targeted interruption of peripheral nociceptive input and sympathetically mediated pain via sustained local anesthetic delivery to the brachial plexus. Continuous peripheral nerve blocks reduce afferent nociception, allow normalization of skin perfusion and temperature, and facilitate early active rehabilitation by providing reliable analgesia without the systemic adverse effects of high dose opioids [6,7].

This report presents an adolescent case of post-traumatic CRPS Type I effectively treated with a combination of subanesthetic ketamine infusion, gabapentin, and a tunneled supraclavicular continuous nerve catheter.

This article adheres to the guidelines laid by the Enhancing the Quality and Transparency of Health Research (EQUATOR) network. Written informed consent for publication and presentation of this case, including the use of clinical images and video material, was obtained from the patient and patient’s parents (Supplementary Materials).

## 2. Case Description

A 15-year-old right-hand dominant female athlete, 71.7 kg, ASA 1, sustained an oblique fracture of the left third metacarpal while performing a gymnastics routine. Closed reduction and percutaneous pinning were performed the following day under general anesthesia, and immobilization was maintained in a short-arm mitten cast. Radiographs at four weeks demonstrated adequate alignment and callus formation, and pins were removed at six weeks.

Approximately three months after surgery, she presented to the hand surgery clinic with a history of persistent, intense burning hand pain accompanied by temperature asymmetry, swelling, and hypersensitivity to light touch since her last clinic visit. She had tried over-the-counter medications and “toughed it out” but did not have relief. She described the pain as continuous, sharp, and aggravated by movement or cold exposure. Examination revealed marked allodynia over the dorsum of the hand, mild coolness compared with the contralateral side, and guarding with active and passive motion. Distal perfusion and strength were preserved, and radiographs confirmed fracture union. The findings fulfilled clinical criteria for CRPS Type I.

Initial outpatient management included occupational therapy focused on graded desensitization, nonsteroidal anti-inflammatory drugs, and acetaminophen as needed. After six weeks, pain persisted at 7/10 on the numeric rating scale, and she continued to experience severe tactile hypersensitivity and limited finger flexion.

The patient was admitted approximately five months after surgery for escalation of care, and the pain service was consulted for managing her pain. On admission, she rated her resting pain 7/10 and exhibited hyperalgesia to light pressure without trophic skin changes or weakness. She reported episodes of excruciating pain, like someone was taking a saw and chopping her fingers off one by one. She was able to flex her fingers at the distal phalanges slightly, but was unable to make a fist due to her pain.

A subanesthetic ketamine infusion was initiated at 7 mg per hour with patient-controlled boluses of 3 mg every 15 min. Acetaminophen 1000 mg and ibuprofen 600 mg were administered orally every 6 h. Gabapentin was started at 300 mg three times daily, and increased to 400 mg three times daily the next day. After 12 h, pain decreased modestly to 5/10, though allodynia persisted, and she could not use her left hand.

**Figure 1:**
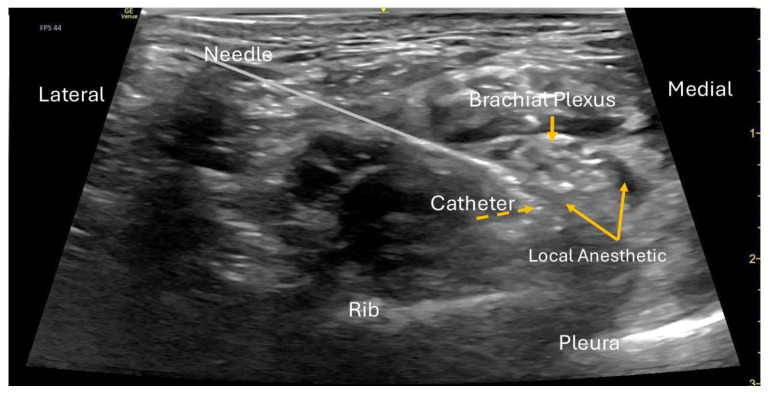
Supraclavicular nerve block with catheter placement.

**Figure 2:**
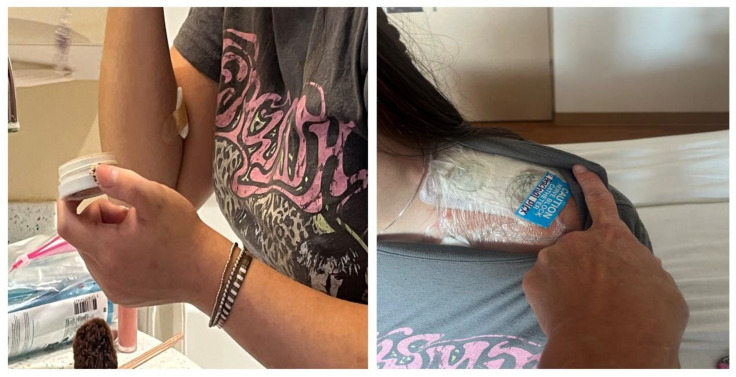
The patient was able to perform left grasp and fine motor movements on day 5 while the supraclavicular catheter was in situ.

On hospital day 2, a left supraclavicular continuous peripheral nerve catheter was placed under ultrasound guidance, while the patient was sedated with ketamine, midazolam, and dexmedetomidine. The procedure was performed under full aseptic precautions and ultrasound guidance using a GE Venue Go system with a high-frequency linear transducer (L4-20t, GE XDclear) to optimize visualization of the brachial plexus cluster in the supraclavicular fossa (Figure 1). After standard sterile preparation and local skin infiltration, an 18-gauge Tuohy-type echogenic needle (Pajunk TuohySono, 18 G × 50 mm) was advanced in-plane from lateral to medial toward the plexus while real-time imaging confirmed correct needle position. After negative aspiration, 1–2 mL of normal saline was injected to confirm perineural spread and to hydro-dissect the plane. A bolus of 0.5% ropivacaine (20 mL) was then administered incrementally, with careful aspiration between aliquots. A 20-gauge flexible catheter (polyamide epidural style catheter, Perifix/B. Braun 20 Ga thread assist guide) was threaded approximately 3 cm beyond the needle tip and confirmed in the correct location. The catheter was tunneled subcutaneously and secured with two chlorhexidine biopatches and a sterile occlusive dressing (Figure 2). The catheter was tunneled to reduce leaking, accidental dislodgement, and infection risk during prolonged outpatient use. Shortly after the placement, the patient experienced complete motor and sensory block in the brachial plexus distribution. Shortly after block placement, the patient developed a mild transient Horner-type effect, consistent with medial and slightly superior spread of local anesthetic to the cervical sympathetic chain or stellate ganglion. The initial symptoms improved within 24 h. During the ambulatory infusion period, she experienced intermittent mild Horner-type symptoms at home while the catheter remained in situ, which fully resolved after catheter removal. Transient Horner-type symptoms after supraclavicular brachial plexus block have been described in prior pediatric and adult case reports, attributed to medial and slightly superior spread of local anesthetic toward the cervical sympathetic chain or stellate ganglion [8,9]. Continuous infusion of ropivacaine 0.2% was initiated on the floor at 7 mL/hour with automated 3 mL boluses every 4 h.

Within 24 h of catheter placement, her pain reduced to 2/10, and by 48 h it was 0/10 at rest. The hand was warm, well-perfused, and could move freely without discomfort. She reported appropriate analgesia. The patient did not require any additional medication administration, and the ketamine infusion was tapered to 3 mg per hour continuous and discontinued on POD (Post Operative Day) 4. Ketamine was discontinued on day 4 because subanesthetic ketamine infusions require inpatient monitoring and cannot be safely continued in the home setting (Table 1).

On hospital day 4, she began active physiotherapy, completing grasp and fine motor exercises without recurrence of pain (Supplementary Materials). The continuous block remained effective, and she tolerated oral intake and ambulation normally. Physiotherapy was initiated on day 4 when analgesia was sufficiently stable and motor function was adequate to allow safe participation without exacerbating pain.

## 3. Ambulatory Phase and Follow-Up

The patient was discharged on hospital day 5 with the catheter in place, connected to an ambulatory infusion pump delivering ropivacaine 0.2 percent at 6 mL per hour with optional 2 mL boluses every hour, and gabapentin 400 mg three times daily.

She went to school for the next four days and was able to resume some of her daily sports activities. The Acute Pain Service conducted daily telephone follow-up. She was pain-free and did not require any additional ropivacaine administration via the pump. The catheter was removed on day 9 when the ambulatory infusion reservoir was nearly empty, coinciding with sustained symptom resolution. She reported intermittent mild Horner-type symptoms at home during the ambulatory infusion period, consistent with expected spread of local anesthetic to the cervical sympathetic chain. These symptoms were not functionally limiting and resolved completely after catheter removal

The catheter was intentionally maintained for nine days to ensure prolonged regional analgesia and sympathectomy during the patient’s early rehabilitation phase. In CRPS, sustained suppression of nociceptive input is beneficial for interrupting central sensitization and allowing consistent engagement in desensitization therapy. Prolonged peripheral nerve catheters, when tunneled and monitored daily, have been reported as safe and effective in carefully selected pediatric patients [6,7,10,11].

Before discharge, the Acute Pain Service provided structured education to the patient and her family regarding catheter care. Education also included pump troubleshooting, recognizing occlusion alarms, and safe pump handling at home. Instructions included daily inspection of the insertion site, maintaining dressing integrity, recognizing signs of infection or catheter dislodgement, proper pump handling, avoidance of moisture, and when to contact the team for urgent concerns. The family demonstrated understanding and performed daily home monitoring with telephone support from the Acute Pain Service. Gabapentin was continued for one week, then tapered over two weeks.

At the two-week follow-up, she was completely pain-free with normal color, temperature, and strength, and she started occupational therapy. At one month, she resumed light athletic activity and remained asymptomatic. At eleven weeks after discharge she remained pain free with full return to sports and daily activities without recurrence (Supplementary Materials). The patient and the family express high satisfaction with the pain control.

## 4. Discussion

This case illustrates the effective use of pharmacologic, interventional, and rehabilitative strategies to achieve rapid remission of adolescent CRPS Type I. The primary goals were adequate pain control, sympathetic modulation, and early restoration of functional hand use. Meeting these goals enabled the patient to participate fully in hand therapy, return to school, and resume light sports soon after discharge.

CRPS arises from abnormal neuroinflammatory signaling, central sensitization, and sympathetic hyperactivity, which together produce severe pain and autonomic changes disproportionate to the initial injury [1]. Pediatric CRPS involves the same mechanisms but is further influenced by psychosocial factors and greater neuroplastic adaptability [2]. The primary therapeutic priority is functional restoration through pain desensitization and graded physical activity [3].

When conservative management fails, multimodal approaches can target several pain-processing pathways. Ketamine interrupts central sensitization and cortical wind-up by blocking NMDA receptor–mediated excitatory transmission [5]. Gabapentin provides complementary analgesia by modulating calcium-channel activity and decreasing presynaptic neurotransmitter release [1]. Continuous peripheral nerve block may provide sustained regional analgesia and sympathetic blockade, minimizing nociceptive input and might contribute to desensitization [6,7]. Despite the immediate effect of the peripheral block, residual central sensitization may persist; therefore, clinical improvement often continues over 24 to 48 h as peripheral nociceptive input is suppressed and central desensitization progresses.

The gradual improvement in pain after catheter insertion may reflects the combined effects of central and peripheral desensitization mechanisms rather than a purely peripheral effect. Similar transient Horner symptoms have been reported after ultrasound-guided supraclavicular and infraclavicular brachial plexus blocks, supporting the known involvement of the cervical sympathetic chain during regional anesthesia of the upper extremity [8,9]. The ketamine infusion was started prior to block placement to reduce central sensitization and continued for four days to maintain NMDA receptor blockade during the early desensitization phase. It was tapered once complete pain relief and functional recovery were achieved.

Physiotherapy was initiated on day 4, after stable analgesia and motor recovery were established, to prevent guarding and stiffness. Early initiation of movement is essential in CRPS rehabilitation but was delayed until the patient could tolerate active participation without discomfort.

The catheter was maintained for 9 days to ensure sustained regional analgesia and sympathetic blockade during the critical phase of rehabilitation. Removal was coordinated with the resolution of allodynia and restoration of full function. In this case, the decision to maintain the continuous supraclavicular catheter for nine days was based on the goal of providing sustained sympathectomy during the early rehabilitative window, when consistent analgesia is essential for interrupting pain amplification and allowing graded functional use [6,7,12].

Although multidisciplinary rehabilitation remains the foundation of pediatric CRPS care, interventional modalities are increasingly important for refractory cases [2,13]. Intensive inpatient rehabilitation has been associated with significant functional improvement, but some patients require additional pain-modulating interventions [13]. Consistent with the WHO biopsychosocial framework, the patient’s care included attention to psychological reassurance, reduction in fear-avoidance behaviors, supervised exposure to functional tasks, and involvement of family support, all of which contributed to normalized hand use and reduction in pain-related anxiety.

In this case, the combination of systemic (ketamine, gabapentin) and regional (supraclavicular nerve block) therapies provided rapid pain relief, enabling early mobilization and facilitating a faster functional reintegration. Similar multimodal strategies in the literature have shown accelerated recovery when pain control allows active participation in physical and occupational therapy. These findings support considering multimodal desensitization as an early adjunct rather than a last-line intervention in refractory CRPS [5,13,14].

Mosquera-Moscoso and colleagues recently reviewed pediatric interventional pain strategies, reporting that ketamine infusions and continuous peripheral nerve catheters are promising adjuncts for CRPS unresponsive to standard therapy. Subanesthetic ketamine use in adolescents has shown favorable analgesic effects with minimal psychomimetic or cardiovascular adverse events when administered with appropriate monitoring [5].

The combined use of systemic and regional techniques may offer synergistic desensitization: ketamine and gabapentin reduce central excitatory signaling, while regional blockade interrupts peripheral nociceptive input, facilitating cortical reorganization and restoration of normal sensorimotor function [15].

### Limitations and Future Directions

This single-patient report cannot establish causal relationships or determine optimal dosing. Pediatric CRPS may remit spontaneously, and controlled data in this population remain limited. Even so, the temporal association between the targeted multimodal interventions and the patient’s complete remission suggests a plausible mechanistic benefit.

Future research should focus on establishing prospective pediatric CRPS registries and developing standardized protocols that integrate pharmacologic and interventional treatments. Defining the optimal duration of continuous nerve blocks, ketamine dosing regimens, and long-term functional outcomes would strengthen evidence for broader clinical application. Although follow-up now extends to eleven weeks, longer term outcome data beyond this period are not available.

## 5. Conclusions

A coordinated multimodal regimen using subanesthetic ketamine infusion, gabapentin, and a prolonged supraclavicular continuous nerve catheter resulted in rapid and sustained remission of refractory adolescent CRPS Type I. Integrating central and peripheral desensitization strategies can facilitate early rehabilitation and support durable functional recovery.

Early, aggressive, and coordinated intervention between surgeons, anesthesiologists, rehabilitation specialists, and pediatric pain services can prevent chronic disability and help children regain independence and quality of life. This case illustrates the effectiveness of well-planned multimodal therapy in safely reducing disease duration and optimizing long-term functional outcomes in pediatric CRPS, thereby adding to the growing evidence supporting multimodal interventional strategies in pediatric CRPS.

## Figures and Tables

**Table 1:** Caption. Pain Scores, Infusion Parameters, and Functional Milestones.

Hospital/Follow-Up Day	Pain Score (0–10)	Ketamine Infusion (mg/h)	Ropivacaine Infusion (mL/h)	Functional Milestones/Clinical Notes
Admission (Day 1)	7	7 mg/h continuous + 3 mg bolus every 15 min	–	Severe pain, allodynia, limited movement
Post-block (Day 2)	2	7 mg/h	7 mL/h + 3 mL every 4 h bolus	Marked relief, improved perfusion; No additional boluses of ketamine used
Day 3	0–1	5 mg/h	7 mL/h + 3 mL every 4 h bolus	Pain-free, began hand therapy No additional boluses of ketamine and ropivacaine were used
Day 4	0	3 mg/h tapered and discontinued.	7 mL/h + 3 mL every 4 h bolus	Active range of motion No additional boluses of ketamine and ropivacaine were used
Discharge (Day 5)	0	Discontinued	6 mL/h +3 mL demand every hour ambulatory pump	Independent activities of daily living. Discharged home
Day 9 (catheter removal)	0	–	–	Catheter removed, no pain or deficit.
2 weeks post-discharge	0	–	–	Full range of motion, back to school
6 weeks post-discharge	0	–	–	Returned to sports, full recovery

## Data Availability

UPMC Children’s Hospital, Department of Anesthesia archived patient’s dataset.

## Supplementary Materials

The following supporting information can be downloaded at: https://www.mdpi.com/article/10.3390/children12121659/s1.
