# Supplementary material for: Effective Adolescent Hand CRPS Type 1 Treatment Using Ketamine, Gabapentin, and Supraclavicular Nerve Block Catheter—A Case Report
Source: Children (Basel). 2025 Dec 7;12(12):1659. doi: 10.3390/children12121659 (PMC12731366; doi:10.3390/children12121659)
Supplement: Supplementary file 1 [file children-12-01659-s001.zip › consent form.zip!/Consent Form for Case Reports.pdf]

Consent Form for Case Reports

Case Report Title

Effective Adolescent Hand CRPS Type 1 Treatment Using Ketamine, Gabapentin, and Supraclavicular Nerve Block Catheter- A Case Report

Principal Investigator: Dr Mihaela Visoiu

UPMC Children's Hospital of Pittsburgh, Department of Anesthesia, Pain Service

412 692 5260

You are being asked to consider allowing Dr. Mihaela Visoiu to use information about your child's pain block to write what is called a case report. Case reports are typically used to share new, unique information experienced by one patient during his/her clinical care that may be useful for other physicians and members of a healthcare team. A case report may be published for others to read and/or presented at a conference. This form explains the purpose of this case report. Please read this form carefully, take the time to make an informed decision, and ask any questions you may have.

This case report informs other physicians about the treatment used to treat your child's hand CRPS type 1.

Your information for this case report includes details about how the blocks were performed, the medication given, pain control measures, ultrasound images, videos, and pictures.

Drs. Mihaela Visoiu must protect your privacy and not disclose your personal information. Your identity will not be disclosed when the case report is published or presented.

Although your personal information collected or obtained will be kept confidential and fully protected by the law, a limited risk associated with this case report could result in a loss of confidentiality under your unique experience.

Participating in this case report will not directly benefit you. However, sharing information with other healthcare professionals may improve the care that others receive in the future.

Allowing your information in this case report will not involve additional costs. You will not receive any compensation.

Taking part in this case report is your choice (voluntary). You may choose not to take part, or you may change your mind at any time. However, once the case report is written and

published, it cannot be withdrawn. Your decision will not result in any penalty or loss of benefits to which you are entitled, including the quality of care you receive.

You will be informed of any new information relating to this case report that may affect you.

Your signature below means that you have read the above information about this Case Report, have had a chance to ask questions to help you understand how your information will be used, and have permitted the use of your data in this case report.

If you have any questions, please call Dr. Mihaela Visoiu at 412-692-5260 (ask the operator to page her).

SUBJECT CONSENT TO PARTICIPATE

Case Report:

Name of Participant: Molly McGovern

Name of Parent: Jerilyn McGovern

Parent Signature : [signature]

Date : 10/9/2025

By signing this form, I confirm that:

- The case report has been fully explained to me and all of my questions have been answered to my satisfaction
- I have been informed of the risks and benefits, if any, of allowing my information to be used in this case report
- I have been informed that is optional to participate in this case report
- I have read each page of this form
- I authorize access to my personal health information (medical record) as explained in this form
- I have agreed to participate in this case report
